# 

**Published:** 1907-06-08

**Authors:** 


### BOOKS RECEIVED.

Longmans, Green and Co.
"Essentials of Chemical Physiology." Sixth edition. By W. D. Halliburton, M.D.

Cassell and Co., Ltd.
"Diseases of Women." Revised edition. By G. E. Herman, M.B.

C. Griffin and Co.
"A Manual of Nursing, Medical and Surgical." 30th edition. By Laurence Humphry, M.D.

J. B. Lippincott Co.
"International Clinics." 17th series. Vol. I.

Hodder and Stoughton.
"Diseases of the Nose and Throat." By J. Bruce Ferguson, M.D. (Medical Epitome Series.)

T. Nelson and Sons.
"The Channings." By Mrs. Henry Wood.

F. S. Turney.
"Cancer: A Working Theory for its Prevention and Cure." By John Shaw, M.D.
"Medical Priestcraft: A National Peril." By John Shaw, M.D.

Eyre and Spottiswoode.
"Printer's Pie."

David Nutt.
"George Buchanan: A Memorial 1506-1906."
"The Early Bird: Why to Get Up Early, and How."

The Charity Organisation Society of New York.
"New York Charities Directory, 1907."

W. Green and Sons, Edinburgh.
"Manual of Midwifery." By W. E. Fothergill, M.D. 4th edit.

Health Resorts Bureau.
"Some Summer Resorts in the South of France." By John W. Potter, F.R.G.S.

H. Kimpton.
"Medical Philosophy." By W. Russell.

H. K. Lewis.
"Lists of Muscles for Electrical Testing."

Percival Marshall and Co.
"Two Hundred Hints for the Laundry." Power Laundry Handbook.

